# Quality and freshness of human bone marrow-derived mesenchymal stem cells decrease over time after trypsinization and storage in phosphate-buffered saline

**DOI:** 10.1038/s41598-017-01315-0

**Published:** 2017-04-24

**Authors:** Tae Hwan Shin, Seungah Lee, Ki Ryung Choi, Da Yeon Lee, Yongman Kim, Man Jeong Paik, Chan Seo, Seok Kang, Moon Suk Jin, Tae Hyeon Yoo, Seong Ho Kang, Gwang Lee

**Affiliations:** 1 Department of Molecular Science and Technology, Ajou University, Suwon, Republic of Korea; 2 Department of Applied Chemistry and Institute of Natural Sciences, Kyung Hee University, Yongin-si, Republic of Korea; 3 Department of Physiology and Department of Biomedical Sciences, Ajou University School of Medicine, Suwon, Republic of Korea; 4 Pharmicell Co., Ltd., Sungnam, Republic of Korea; 5 College of Pharmacy, Sunchon National University, Suncheon, Republic of Korea; 6 Hanyang University School of Medicine, Seoul, Republic of Korea; 7 Biological Sciences, Ajou University, Suwon, Republic of Korea

## Abstract

Human bone marrow-derived mesenchymal stem cells (hBM-MSCs) have been studied for their therapeutic potential. However, evaluating the quality of hBM-MSCs before transplantation remains a challenge. We addressed this issue in the present study by investigating deformation, the expression of genes related to reactive oxygen species (ROS) generation, changes in amino acid profiles, and membrane fluidity in hBM-MSCs. Deformability and cell size were decreased after storage for 6 and 12 h, respectively, in phosphate-buffered saline. Intracellular ROS levels also increased over time, which was associated with altered expression of genes related to ROS generation and amino acid metabolism. Membrane fluidity measurements revealed higher Laurdan generalized polarization values at 6 and 12 h; however, this effect was reversed by *N*-acetyl-l-cysteine-treatment. These findings indicate that the quality and freshness of hBM-MSCs is lost over time after dissociation from the culture dish for transplantation, highlighting the importance of using freshly trypsinized cells in clinical applications.

## Introduction

Mesenchymal stem cells (MSCs) have potential applications in stem cell therapy for the treatment of myocardial infarction, spinal cord injury, ischemic diseases, and neurodegenerative disorders^1, 2^. In particular, bone marrow-derived (BM-)MSCs are readily obtained and have favorable characteristics such as a high degree of plasticity, immunosuppressive activity, and ease of handling *in vitro* ^3^. Human (h)BM-MSCs are considered a promising clinical tool for the treatment of myocardial infarction, ischemic stroke, multiple system atrophy, and alcoholic cirrhosis^4–7^.

About 100 million autologous hBM-MSCs cultured in dishes are required for transplantation^4–6^. It is imperative that the quality and freshness of the cells be preserved prior to transplantation by minimizing stress and damage^8^. However, after dissociation from the culture dish, hBM-MSCs are serum-starved before transplantation into patients^8–10^, which can cause stress to the cells. In our previous study, we found that gene expression levels were perturbed and the rate of cell death was increased in hBM-MSCs over time^8^.

Cell deformability is a label-free biomarker used to assess various cell conditions such as metastatic potential, cell cycle stage, degree of differentiation, and leukocyte activation; it reflects physical changes in cellular components including the membrane, cytoskeleton, and nucleus^11^. For instance, the deformability of red blood cells (RBCs) in patients suffering from sickle-cell disease, malaria, or diabetes differs from that of healthy cells^12^. Moreover, oxidative stress and lipid peroxidation reduce RBC deformability by disrupting the cell membrane^13^. Deformability, which can be measured using microfluidics approach, is used as a criterion for evaluating the quality of hBM-MSCs; indeed, it was found to be significantly reduced by storage for 24 h in phosphate-buffered saline (PBS)^14^. However, it is unknown how cellular damage alters cell deformability. We previously showed that at earlier time points—*i.e*., after 6 or 12 h in PBS—the viability and deformability of hBM-MSCs were reduced and cell morphology and gene expression were altered^8^.

Amino acid profiling can provide information on perturbations in cellular homeostasis, including activation of apoptosis and the immune response and generation of reactive oxygen species (ROS)^15^. Amino acids are converted to organic acid *via* glucogenic and ketogenic pathways and are closely linked to energy generation in mitochondria through the tricarboxylic acid cycle^16^. Amino acid profiling in hBM-MSCs can reveal cellular responses to malnutrition resulting from reduced proteasomal activity as well as the use of amino acids as an energy source^15^.

Polyunsaturated phospholipids, glycolipids, and cholesterol are targets of ROS in the peroxidation of plasma membrane lipids^17^. Oxidative cleavage of polyunsaturated phospholipids generates malondialdehyde, acrolein, and 4-hydroxynonenal as by-products^18^ and depletes unsaturated phospholipid and cholesterol in the membrane, leading to loss of membrane fluidity and permeability^19^ that can affect physiological functions^20^.

Loss of membrane fluidity is an indicator of membrane dysfunction. Several studies have reported that membrane fluidity is decreased by lipid peroxidation^19, 21^. Fluidity can be measured using Laurdan, a polarity-sensitive membrane probe that exhibits a 60-nm spectral shift from disordered to ordered bilayer phases^22^. However, the relationship between cell deformability and membrane fluidity remains poorly understood, despite the fact that these characteristics provide critical information on the quality of cells, including stem cells.

In this study, we investigated changes in hBM-MSC deformability by microfluidic-based measures of cell stretching. In addition, we examined alterations in gene expression and amino acid profiles, ROS generation, and changes in membrane fluidity in cells maintained in PBS for short periods. Our results suggest that the quality of hBM-MSCs decreases over time, which should be taken into consideration when these cells are used for therapeutic applications.

## Results

### hBM-MSCs deformability and cell size decrease over time

**Figure 1:**
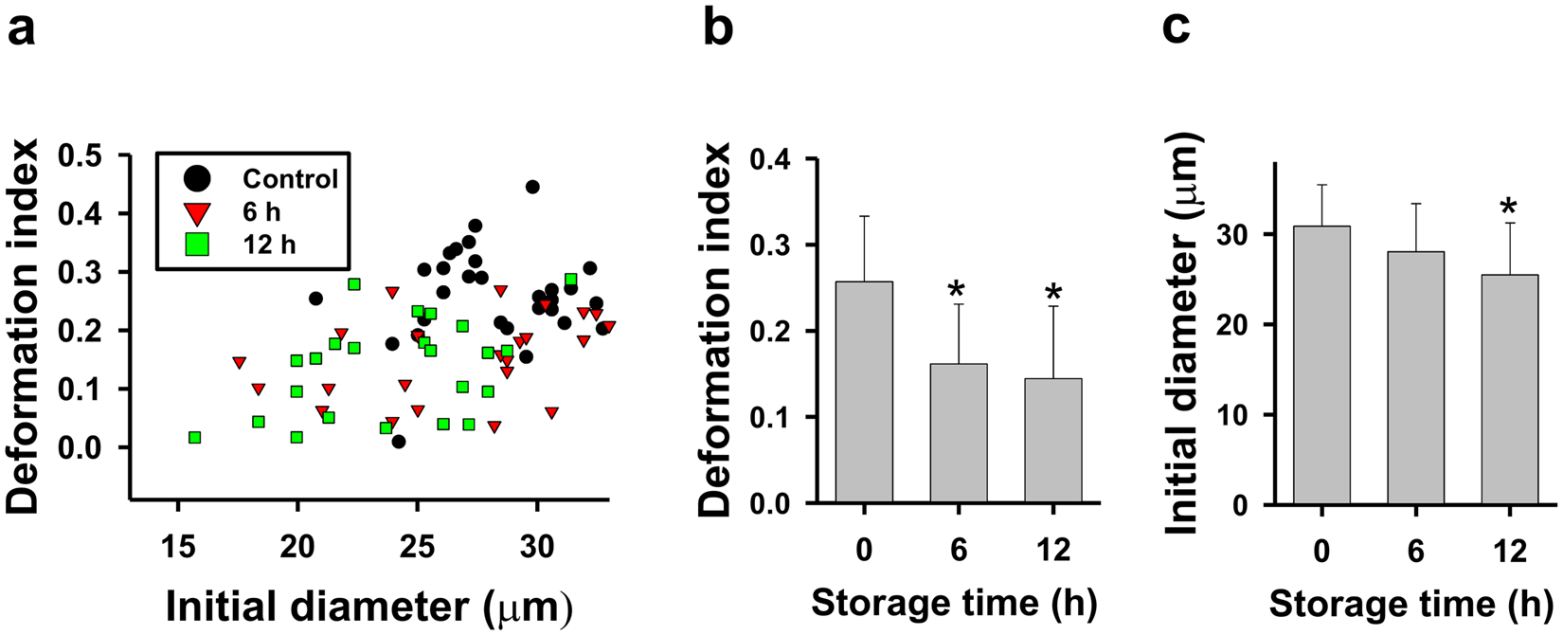
Changes in deformability and cell size of hBM-MSCs stored in PBS. (**a**,**b**) Cell deformability and (**a**,**c**) cell size were measured using a four-walled polydimethoxysilane microfluidic device at a flow rate of 160 μl/h. Data represent mean ± SD (N > 20). *P < 0.05 *vs*. 0 h (control).

The change in the deformability of hBM-MSCs was measured hourly for 12 h by microfluidics. Deformability decreased over time, with a significant difference at 6 and 12 h relative to the 0 h time point (Fig. 1a,b and Supplementary Fig. 1). Cell size also decreased over time, with a significant difference observed at 12 h (Fig. 1a,c).

### Expression of ROS-related genes and ROS generation change over time in hBM-MSCs

**Figure 2:**
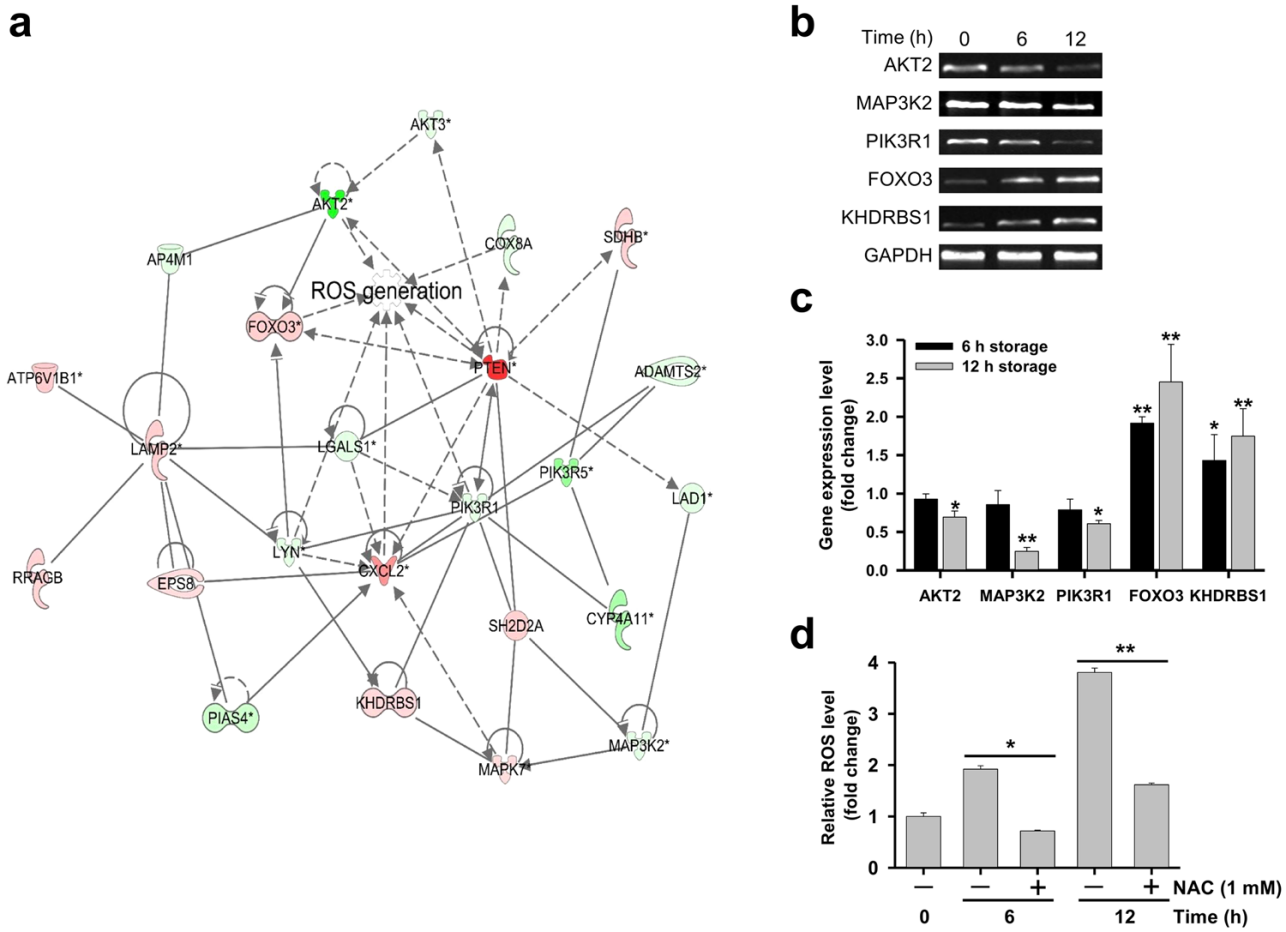
Changes in ROS-related gene expression and ROS generation in hBM-MSCs over time. (**a**) An ROS-related gene network was constructed algorithmically by IPA. Red and green areas indicate up- and downregulated genes, respectively. Differentially expressed genes were identified by microarray analysis without fold-change cut-off. (**b**) Semi-quantitative RT-PCR detection of ROS-related genes (*AKT2*, *MAP3K2*, *PIK3R1*, *FOXO3*, and *KHDRBS1*) in hBM-MSCs after 6 or 12 h of storage in PBS. Glyceraldehyde 3-phosphate dehydrogenase (*GAPDH*) served as an internal control. Bands were cropped from Supplementary Fig. 2. (**c**) ROS-related gene expression in hBM-MSCs stored in PBS for 6 or 12 h, as determined by qPCR. Relative gene expression levels were normalized to that of GAPDH and compared to the level at 0 h (control). (**d**) Evaluation of intracellular ROS generation after 6 or 12 h using DCFH-DA. The intensity of non-oxidized DCFH-DA was used as a blank. Data represent mean ± SD of three independent experiments. *P < 0.05, **P < 0.001 *vs*. 0 h (control).

The expression of 24 ROS-related genes in hBM-MSCs was altered after 6 and 12 h of culture in PBS (Supplementary Table 1). Ingenuity Pathway Analysis (IPA) was used to evaluate the relationships between genes based on microarray data. The levels of genes related to ROS generation were significantly altered (Fig. 2a): semi-quantitative reverse transcription (RT-)PCR (Fig. 2b and Supplementary Fig. 2) and quantitative real-time (q)PCR (Fig. 2c) analyses revealed that V-akt murine thymoma viral oncogene homolog 2 (*AKT2*), mitogen-activated protein kinase kinase kinase 2 (*MAP3K2*), and phosphoinositide-3-kinase regulatory subunit 1α (*PIK3R1*) were downregulated whereas Forkhead box O3 (*FOXO3*) and KH domain-containing, RNA-binding, signal transduction-associated 1 (*KHDRBS1*) were upregulated after 12 h. We also assessed intracellular ROS generation in hBM-MSCs over time by 2′,7′-dichlorodihydrofluorescin diacetate (DCFH-DA) staining. ROS levels increased after 6 and 12 h in PBS, an effect that was inhibited in the presence of the ROS scavenger N-acetyl-l-cysteine (NAC) (Fig. 2d).

### Amino acid profiles change over time in hBM-MSCs

**Figure 3:**
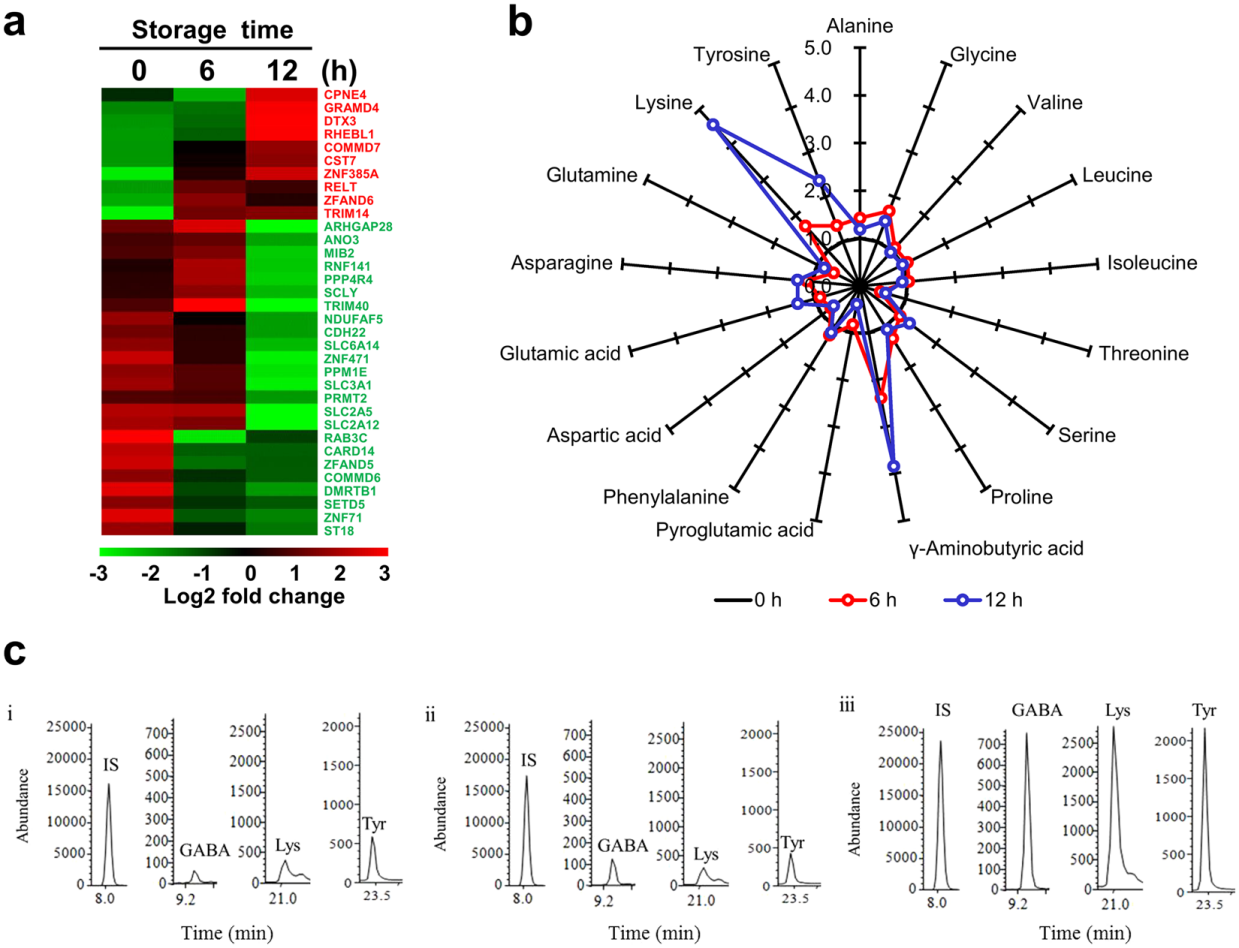
Changes in transcriptome and amino acid profiles in hBM-MSCs stored in PBS for 6 and 12 h. (**a**) Heat map of genes with altered expression; 34 genes related to amino acid metabolism were differentially expressed at 6 and 12 h according to a microarray analysis. Red and green areas indicate up- and downregulated genes, respectively. (**b**) Star plot showing altered levels of 17 amino acids after 6 and 12 h of incubation in hBM-MSCs. Data are based on percentage mean composition levels of 17 amino acids after normalization to the corresponding values in the control group. Three independent experiments were performed. Selected-ion monitoring (SIM) chromatograms of GABA, lysine, and tyrosine in (i) 0 h control (ii) 6 h group (iii) 12 h group. IS, Internal standard (norvaline).

ROS generation is closely related to the amino acid content of cells, since their efflux and influx are controlled by mitochondria, which are a source of ROS^23, 24^. We carried out a microarray expression analysis using MultiExperiment Viewer (MeV) software to cluster 34 genes related to amino acid metabolism. In hBM-MSCs stored for 12 h in PBS, the expressions of amino acid metabolism-related genes were significantly changed as compared to the 0 h time point (Fig. 3a).

The effect of storage time on cellular amino acid composition was investigated in hBM-MSCs stored for 6 and 12 h by gas chromatography–mass spectrometry (GC-MS), with values normalized to the mean levels in the control group (Supplementary Table 2). A plot of these values showed that the levels of lysine, tyrosine, and γ-aminobutyric acid (GABA) increased with storage time, with the highest levels observed at 12 h (Fig. 3b). The representative SIM chromatograms revealed that GABA, lysine, and tyrosine of 12 h group were considerably altered compared to 6 h group (Fig. 3c).

### Gene co-expression network and amino acid profiles in hBM-MSCs

**Figure 4:**
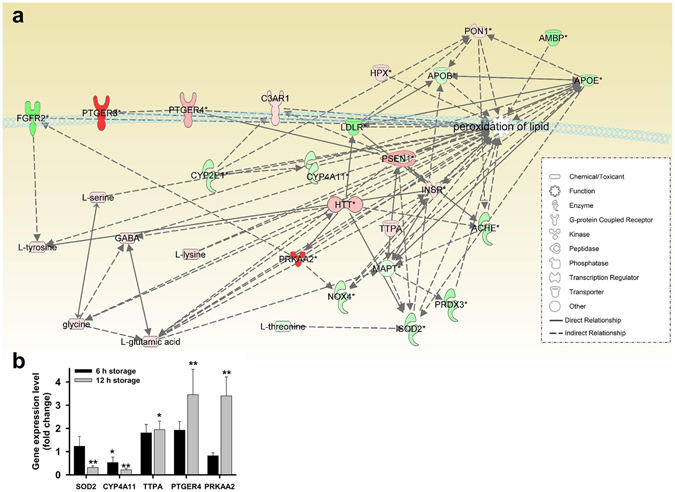
Bioinformatics analysis of microarray and amino acid profiles in hBM-MSCs stored in PBS for 12 h. (**a**) Lipid peroxidation-related genes and amino acid network were constructed algorithmically by IPA. Red and green areas indicate up- and downregulated genes, respectively. Differentially expressed genes obtained from microarray data (genes with >3-fold change) are shown. (**b**) qPCR analysis of lipid peroxidation-related gene expression in hBM-MSCs stored in PBS for 6 and 12 h. Data represent mean ± SD of three independent experiments. *P < 0.05, **P < 0.001 *vs*. 0 h (control).

A gene co-expression network was constructed from microarray and amino acid profiling data by IPA. The data showed increased lipid peroxidation at 12 h (Fig. 4a, Supplementary Table 3) as compared to the 6 h time point (Supplementary Fig. 3). GABA, glutamate, lysine, serine, and glycine were directly related to lipid peroxidation along with 22 genes at 12 h. The expression of lipid peroxidation-related genes was quantified by qPCR (Fig. 4b), which showed that superoxide dismutase 2 (*SOD2*) and cytochrome P450, family 4 subfamily A polypeptide 11 (*CYP4A11*) were downregulated whereas tocopherol α transfer protein (*TTPA*), prostaglandin E receptor 4 (*PTGER4*), and protein kinase, AMP-activated, alpha 2 catalytic subunit (*PRKAA2*) were upregulated in the 12 h relative to the 0 h group.

### Membrane fluidity decreases and lipid peroxidation increases over time in hBM-MSCs

**Figure 5:**
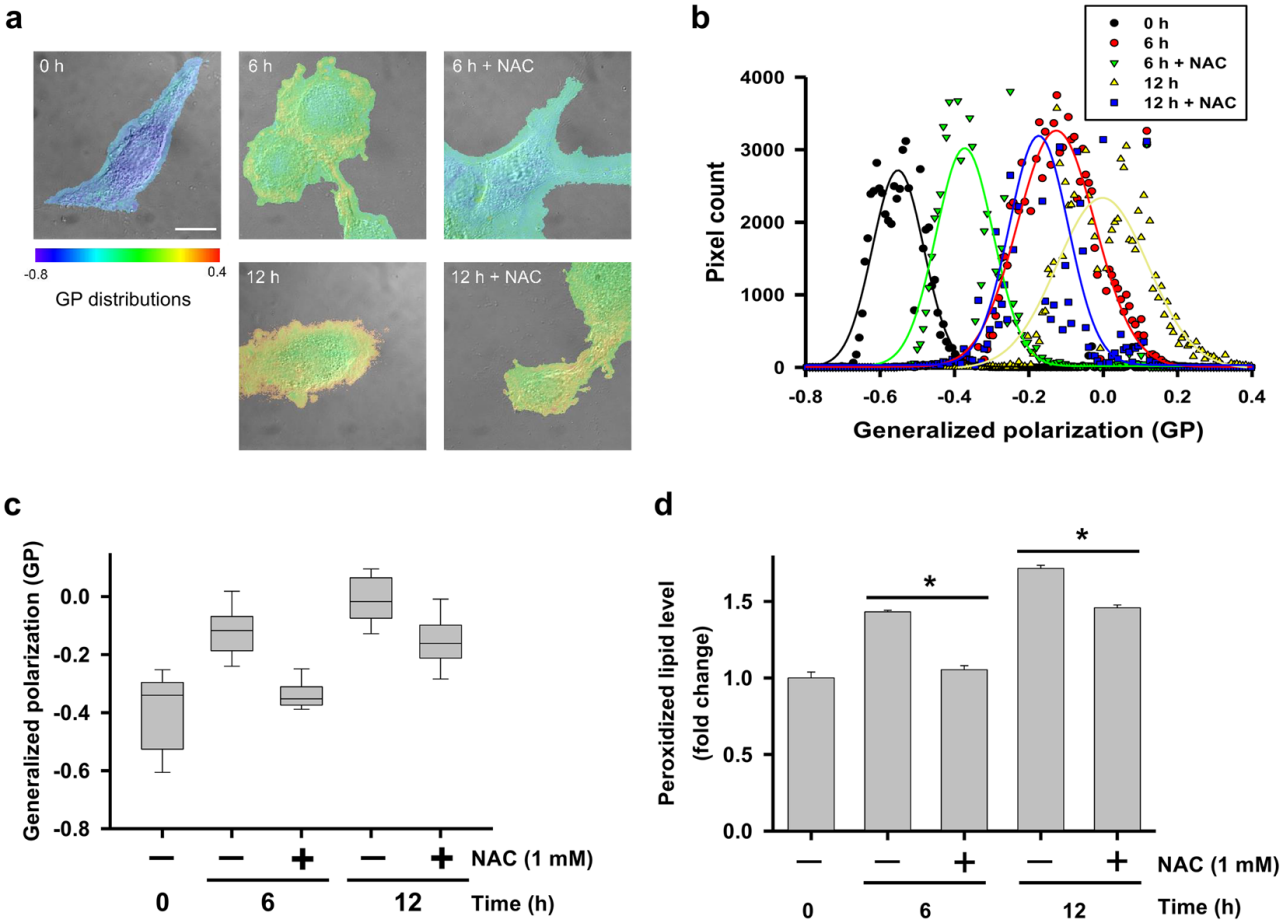
Laurdan GP images, GP frequency distributions, and evaluation of peroxidized lipids in hBM-MSCs stored in PBS for 6 and 12 h. (**a**) Merged DIC and TIRFM images of control hBM-MSCs. GP distributions were ranged from −0.8 to 0.4. Scale bar = 10 µm. (**b**) GP frequency distributions of hBM-MSCs. GP values of each pixel are represented as dots and were fitted to Gaussian distributions. (**c**) Total GP values. Data represent the mean ± SD of three independent experiments (n = 10). (**d**) Evaluation of peroxidized lipids using ferrous thiocyanate. The intensity of non-peroxidized lipid was used as a blank. Data represent mean ± SD of three independent experiments. *P < 0.05.

hBM-MSCs membrane fluidity was determined based on Laurdan generalized polarization (GP) values and analyzed by total internal reflection fluorescence microscopy (TIRFM). The number of high-GP areas on the hBM-MSCs surface—corresponding to rigid domains—increased over time (Fig. 5a); increases in GP over time were suppressed in the presence of NAC, with a GP scale of −0.8 to 0.4. GP frequency distribution values were subtracted from corresponding values in control cells to obtain frequency difference curves (Fig. 5b) and total mean GP values (Fig. 5c). The difference values increased with storage time, an effect that was inhibited in the presence of NAC. A similar trend was observed in the relative levels of peroxidized lipid (Fig. 5d). These results indicate that rigid regions in hBM-MSC plasma membrane are increased over time *via* lipid peroxidation.

## Discussion

The present study used a combination of microfluidics-based assessment of cell deformability, transcriptomics and metabolomics analyses, and differential interference contrast (DIC)-TIRFM measurement of membrane fluidity to evaluate the quality of hBM-MSCs stored in PBS over time. Our results indicate that minimizing storage time and blocking ROS generation are essential for preserving hBM-MSC quality for clinical applications.

The deformability of hBM-MSCs was dramatically altered after incubation in PBS for 24 h^14^. In this study, we observed a decrease in deformability at earlier time points (6 and 12 h). Moreover, cell size also decreased over time while morphology was altered. These results suggest that hBM-MSCs became stiff with increasing time in PBS. This is supported by the observed reduction in hBM-MSC viability reported in our previous study^8^ and the fact that erythrocytes with lipid abnormalities and low deformability are more vulnerable to osmotic stress and have reduced capacity for passing through vessel walls^25^.

A decrease in cell size, as observed here in hBM-MSCs stored in PBS, is associated with reductions in cell contents including DNA, proteins, and lipids^26–28^. Cell size is also related to autophagy and is regulated by mammalian target of rapamycin 1/2 activity, which is controlled by Ras-related C3 botulinum toxin substrate 1 (*Rac1*)^29^. We found that autophagy in hBM-MSCs increased with time, which was reversed by NAC treatment (Supplementary Fig. 4). We speculate that ROS are the main factors leading to autophagy in hBM-MSCs, although additional studies are needed to confirm this possibility.

Changes in amino acid profiles of cells reflect dysregulation of homeostasis^30^, which can perturb various cellular functions. Free amino acids such as tryptophan, tyrosine, histidine, and cysteine can be directly attacked by ROS^31^. We found that the levels of amino acids related to lipid peroxidation were altered in hBM-MSCs after 6 and 12 h of storage in PBS. GABA suppresses Ca^2+^ release, ROS production, and lipid peroxidation upon neuronal injury, both *in vivo* and *in vitro* ^32^. Glycine attenuates superoxide anion radical release in the presence of nicotinamide adenine dinucleotide phosphate and decreases protein carbonyl and lipid peroxidation by increasing the levels of glutathione synthetase and consequently, of glutathione^33^. ROS generation is increased by accumulation of basic amino acids such as arginine, ornithine, and lysine in the mitochondrial membrane, which induces mitochondria-dependent cell death *via* aberrant ubiquitination^34^. Thus, the response to ROS generation under serum starvation is closely related to changes in cellular amino acid composition.

Deficiency in SOD2—a major antioxidant enzyme—leads to lipid peroxidation *via* apolipoprotein B activation in a mouse model^35^. CYP4A proteins located in mitochondria also modulate the antioxidant pathway^36^. Genes encoding SOD2 and CYP4A were downregulated in our study. Altered expression of the α-tocopherol transporter protein TTPA and prostaglandin E2 receptor PTGER4^37^ could affect the cellular response to lipid peroxidation and reduce membrane fluidity; our data suggest that such a reduction is responsible for the decrease in hBM-MSC deformability over time.

In the present study, we evaluated the effects of the common ROS scavenger NAC on hBM-MSCs over time. NAC is used as an inhibitor of ROS-induced apoptosis by oxidizing its own thiol group when used at a low concentration (<5 mM)^38^. However, NAC also induces apoptosis via inhibition of NF-κB when used at high concentrations (>20 mM)^39^. In this study, ROS generation and lipid peroxidation were reduced in presence of 1 mM NAC in hBM-MSCs overtime. In addition, NAC also restored cell deformability in hBM-MSCs over time, although this effect was not statistically significant (data not shown). Thus, inhibition of ROS and the addition of other additives for maintaining cell “freshness” with proper concentrations will be helpful for the establishment of effective BM-MSC therapies.

Differentiation capacity is the essential function of BM-MSC for therapies^10^. We analyzed osteogenic and adipogenic potentials with hBM-MSCs before and after exposure to PBS and NAC-treated hBM-MSCs. In osteogenic differentiation condition, there were no significant differences in differentiation capacities of hBM-MSCs. That is, mineral deposition and alkaline phosphatase activity of differentiated cells were not significantly different (Supplementary Fig. 5a,b). In addition, lipid deposition and mRNA expression levels of adipocyte specific marker genes, peroxisome proliferator-activated receptor (*PPARγ*) and complement factor D (*Adipsin*), were also not significantly different in adipogenic differentiation condition (Supplementary Figs 5c,d and 6). Thus, we suppose that the initial differences of PBS-stored hBM-MSCs and NAC-treated hBM-MSCs were faded out within the period for hBM-MSCs differentiation and shown no differences at the endpoint of differentiation.

In conclusion, hBM-MSCs used in clinical applications should be prepared as quickly as possible after disassociation from the culture dish and treated with an ROS blocker in order to preserve cell quality and ensure a successful outcome following transplantation.

## Materials and Methods

### Cell culture

hBM-MSCs were purchased from PromoCell (Heidelberg, Germany) from one donor (a 65-year-old Caucasian man) and were cultured as described in our previous study^8^. Briefly, the cells were rinsed with PBS and resuspended in low-glucose Dulbecco’s modified Eagle’s medium containing 10% fetal bovine serum and 1% penicillin/streptomycin (all from Gibco, Grand Island, NY, USA). Cells were harvested after six passages as we have previously reported^8^. Expression of hBM-MSC surface markers cluster of differentiation (CD)105 and CD73 was detected by flow cytometry^4^; cells were characterized as having high expression (~99%) of these positive markers and low expression (~1%) of the negative markers CD34 and CD45 (Supplementary Fig. 7). Approximately 10^6^ cells from each fraction were centrifuged at 500 × *g* for 5 min, washed three times in PBS, and then incubated in PBS for 6 or 12 h.

### Microfluidics measurement of cell deformation index

The deformability of hBM-MSCs was analyzed as previously described^14^. Briefly, a four-walled polydimethoxysilane microfluidic device fabricated using a standard photolithography method was used to measure deformation, with the longest and shortest length of the cell at the stagnation point of the cross-slot channel while monitoring stretch in the extensional flow. A 6.8 wt% polyvinylpyrrolidone (PVP) solution in PBS was used as the mobile fluid, with viscosity = 90 cP and relaxation time = 9.4 × 10^−4^ s.

### qPCR

The expression of ROS- and lipid peroxidation-related genes was detected by qPCR using the RealMOD SYBR Green real-time PCR kit (Intron) with gene-specific primer pairs (Supplementary Tables 5 and 6) on a Rotor Gene-Q system (Qiagen, Valencia, CA, USA). Reaction conditions were as follow: 95 °C for 5 min, followed by 50 cycles of 95 °C for 5 s and 60 °C for 30 s. The threshold/quantification cycle (Ct/Cq) value was determined at the point where the detected fluorescence was statistically higher than the background level. PCR products were analyzed based on a melting curve constructed using Rotor-Gene 1.7 software (Qiagen). PCR reactions were prepared as independent triplicate samples. The relative quantification of target gene expression was calculated by the 2^−ΔΔCt^ method.

### GC–MS

Each of the amino acid content was analyzed by GC–MS as described in our previous report^40^. Briefly, GC–MS analyses in both scan and SIM modes were carried out on a model 6890 N gas chromatograph (Agilent Technologies, Santa Clara, CA, USA) interfaced with a model 5975B mass-selective detector (70 eV, electron impact ionization mode; Agilent Technologies).

### Microarray and amino acid profiling

Changes in gene expression in hBM-MSCs were examined using the Affymetrix system (Istech, Ilsan, Korea) in conjunction with the Human U133 Plus 2.0 50 K microarray containing 54,675 probes. Differences in data distribution were analyzed with GenPlex 3.0 software as described in our previous report^8^. Biological pathways and functions were determined using the IPA web-based bioinformatics software (Qiagen). A 3-fold change in gene expression of 6 and 12 h stored hBM-MSCs was used as a cut-off value for genes with significant changes in expression in comparison with 0 h control.

### Measurement of membrane fluidity

Changes in membrane fluidity were measured using Laurdan and a homemade DIC-TIRFM system, as described in our previous report^41^. The protocol has been described elsewhere^42^. Briefly, cells were seeded on cover slips (no. 1 thickness, 0.13–0.16 mm) and incubated in PBS for 6 or 12 h in the presence or absence of 1 mM NAC. For Laurdan staining, cells were incubated with medium containing 10 µM dye at 37 °C/5% CO_2_ for 2 h, then washed twice with PBS and fixed with Cytofix buffer (BD, San Jose, CA, USA). Cover slips with cells were mounted on glass slides with Prolong Gold Antifade mounting medium (Molecular Probes). Changes in Laurdan fluorescence were visualized with a 100× oil iris objective lens (NA = 0.6–1.3, UPLANFLN, Olympus Optical Co., Ltd., Tokyo, Japan) and images were captured with a electron-multiplying cooled charge-coupled device (EMCCD) camera (QuantEM 512SC; Photometrics, Tucson, AZ, USA). Excitation and emission wavelengths were 405 and 420 nm, respectively, with a 473 nm bandpass filter (resolution: ±5 nm). Membrane fluidity was determined with the formula generalized polarization (GP) = (Intensity_420 nm_ − Intensity_473 nm_)/(Intensity_420 nm_ + Intensity_473 nm_). Merged DIC/GP images were generated using ImageJ software. Gaussian distributions were generated using the nonlinear fitting algorithm in SigmaPlot v.10.0 software (Systat, San Jose, CA, USA).

### Statistical analysis

The results were analyzed by one-way analysis of variance (ANOVA) using IBM-SPSS software (IBM Corp., USA). Differences with P values of less than 0.05 were considered significant.

## Electronic supplementary material

Supplementary information
