# Supplementary material for: Quality and freshness of human bone marrow-derived mesenchymal stem cells decrease over time after trypsinization and storage in phosphate-buffered saline
Source: Sci Rep. 2017 Apr 24;7:1106. doi: 10.1038/s41598-017-01315-0 (PMC5430841; doi:10.1038/s41598-017-01315-0)
Supplement: Supplementary file 1 — Supplementary information [file 41598_2017_1315_MOESM1_ESM.pdf]

Supporting Information

Quality and freshness of human bone marrow-derived mesenchymal stem cells decrease over time after trypsinization and storage in phosphate-buffered saline

Tae Hwan Shin[1,3#], Seungah Lee[2#], Ki Ryung Choi[3,4], Da Yeon Lee[3], Yongman Kim[4], Man Jeong Paik[5], Chan Seo[5], Seok Kang[6], Moon Suk Jin[7], Tae Hyeon Yoo[1], Seong Ho Kang[2*], Gwang Lee[3]*

[1]*Department of Molecular Science and Technology, Ajou University, Suwon, Republic of Korea*

[2]*Department of Applied Chemistry and Institute of Natural Sciences, Kyung Hee University, Yongin-si, Republic of Korea*

[3]*Department of Physiology and Department of Biomedical Sciences, Ajou University School of Medicine, Suwon, Republic of Korea*

[4]*Pharmicell Co., Ltd., Sungnam, Republic of Korea*

[5]*College of Pharmacy, Sunchon National University, Suncheon, Republic of Korea*

[6]*Hanyang University School of Medicine, Seoul, Republic of Korea*

[7]*Biological Sciences, Ajou University, Suwon, Republic of Korea*

[#]: These authors contributed equally to this work.

[*]Co-corresponding author: Seong Ho Kang, Professor Department of Applied Chemistry and Institute of Natural Sciences, Kyung Hee University, Yongin-si, Gyeonggi-do 446-701 Republic of Korea. Tel : +82 31 201 3349, email : shkang@khu.ac.kr

*Corresponding author: Gwang Lee, Professor, Department of Physiology, Ajou University School of Medicine, 164, World cup-ro, Yeongtong-gu, Suwon 16499, Republic of Korea. Tel : +82-31-219-4554, fax: +82-31-219-5049, e-mail : glee@ajou.ac.kr

Materials and Methods

Total RNA isolation

Total RNA was isolated from hBM-MSCs using RNAzol B (Tel-Test, Friendswood, TX, USA). Briefly, 2×10^6 cells were harvested in RNAzol B solution, followed by addition of chloroform and incubation for 5 min on ice. Cells were then treated with isopropyl alcohol to precipitate total RNA. Pellets were washed in 70% ethanol followed by air drying, and total RNA was dissolved with RNase-free water. RNA purity was determined with optical density values of 1.8–2.0 at wavelength ratio of 260/230 and 260/280, using spectrophotometry (Eppendorf, Hamburg, Germany).

Evaluation of intracellular ROS levels

Intracellular ROS was evaluated by DCFH-DA staining (Cell Biolabs, San Diego, CA, USA) according to the manufacturer's protocol. Briefly, cells were resuspended in dye solution (10 μM DCFH-DA in PBS) and incubated in 37°C/5% CO_2 for 1 h. Samples were washed twice with PBS, and fluorescence was measured with a Gemini EM fluorescence microplate reader (Molecular Devices, Sunnyvale, CA, USA) at 480 nm excitation/530 nm emission.

Semi-quantitative RT-PCR

ROS-related gene expression and adipocyte specific marker gene expression were detected by semi-quantitative RT-PCR using gene-specific primer pairs (Supplementary Table 4, 7). To prepare cDNA, total RNA (1 μg) was reverse transcribed using the Power cDNA Synthesis Kit (Intron, Sungnam, Korea) and oligo dT primers on a Thermocycler T3000 PCR system

(Whatman Biometra, Gottingen, Germany). PCR reactions were carried out using 100 ng cDNA, 5 pmoles of each primer, and Biomix (Biolines, Taunton, MA, USA). Each reaction was subjected to melting point analysis to confirm that a single product had been amplified. Products were separated on 2% agarose gel and visualized by ethidium bromide staining.

Evaluation of autophagy induction

Autophagic vacuoles were stained to evaluate autophagy induction using monodansylcadaverine (MDC) as manufacturer's instruction (Cayman Chemical, MI, USA). Briefly, after 6 and 12 h PBS-incubation, hBM-MSCs were plated at a density of 5×10^4 cells/well in a 96 well plate and centrifuged at 400 g for 5 min at room temperature. The cells were then washed with cell-based assay buffer (Cayman Chemical, MI, USA), incubated with 100 µl of Cell-Based MDC solution (Cayman Chemical, MI, USA) for 10 min at 37°C, and examined under Axio 200M Zeiss fluorescent microscope (Carl Zeiss Inc., Göttingen, Germany). The cells were transferred to 96-well black plate and were measured for fluorescence microplate reader at 335/512 nm (excitation/emission).

Evaluation of lipid peroxidation

Peroxidized unsaturated lipids were quantified using a kit (Cayman Chemical, Ann Arbor, MI, USA) according to the manufacturer's instructions. Briefly, cells were detached from the culture dish and incubated in PBS for 6 or 12 h in the presence or absence of 1 mM NAC. After two washes with PBS, the cells were transferred to glass test tubes. Lipids were extracted with methanol saturated with crystalline solid and ice-cold chloroform. After five rounds of centrifugation (1500 × *g* at 0°C), the bottom chloroform layer was collected and mixed at a 9:1

ratio with a solution containing 2.25 mM ferrous sulfate, 0.1 M hydrochloric acid, and 1.5% ammonium thiocyanate in methanol, followed by incubation at room temperature for 5 min. Ferric ions were produced in the reaction and peroxidized unsaturated lipids were detected using thiocyanate as a chromogen. Absorbance at 500 nm was measured on a microplate reader (Molecular Devices) in a quartz cuvette.

Evaluation of differentiation capacity

hBM-MSCs were plated at a density of 5,000 cells/cm^2 after PBS-storage for 6 or 12 h in the presence or absence of 1 mM NAC, and then cultured at 37 ^{o}C in humidified atmosphere of 5% CO_2 in condition medium of adipogenesis (StemPro Adipogenesis Differentiation Kit, Invitrogen, CA, USA) or osteogenesis (StemPro Osteogenesis Differentiation Kit, Invitrogen, CA, USA) induction for 2 weeks, respectively. To maintain the cells in a differentiation, condition medium was changed every 72 h.

To assess osteogenic differentiation of hBM-MSCs, Alizarin Red S staining was performed after 2 weeks of differentiation. Briefly, cells were fixed with Cytofix buffer (BD, San Jose, CA, USA), and then rinsed with distilled water and stained with 2% Alizarin Red S (Sigma-Aldrich) in the dark for 45 min. Cells were washed 4-times with distilled water and PBS was added. Images were taken Axio 200FL Zeiss microscope equipped with AxioCam ICc5 (Carl Zeiss Inc., Göttingen, Germany). Alkaline phosphatase activity of osteogenic differentiated cell was evaluated after 2 weeks of culture in osteogenic differentiation media. Alkaline phosphatase activity assay was performed using pNPP-based alkaline phosphatase Staining Kit (Sigma-Aldrich). Absorbance at 405 nm was measured on a microplate reader (Molecular Devices).

To evaluate adipogenic differentiation of hBM-MSCs, Oil Red O staining was performed after 2 weeks of differentiation. Briefly, cells were fixed with Cytofix buffer (BD, San Jose, CA, USA), and then rinsed with distilled water and incubated in 60% isopropanol for 5 min. Cells were stained with 0.18% Oil Red O dissolved in 60% isopropanol for 20 min, and then washed 4-times with distilled water. Images were taken Axio 200FL Zeiss microscope equipped with AxioCam ICc5 (Carl Zeiss Inc., Göttingen, Germany).

Supplementary Table 1. Ingenuity Pathway Analysis-based profiles of ROS generation-related genes in hBM-MSCs cells over time

Entrez gene name	Symbol	Affymetrix ID	Location	Signal (fold change)[a]	
				6 h	12 h
ADAM metallopeptidase with thrombospondin type 1 motif 2	*ADAMTS2*	214454_at	Extracellular space	−1.08	−1.17
v-Akt murine thymoma viral oncogene homolog 2	*AKT2*	203809_s_at	Cytoplasm	−5.79	−24.49
v-Akt murine thymoma viral oncogene homolog 3	*AKT3*	219393_s_at	Cytoplasm	−2.38	−2.39
Adaptor-related protein complex 4 mu 1 subunit	*AP4M1*	209837_at	Cytoplasm	−1.28	−1.18
ATPase H+ transporting V1 subunit B1	*ATP6V1B1*	1554847_at	Cytoplasm	−1.72	1.53
Cytochrome c oxidase subunit 8A	*COX8A*	201119_s_at	Cytoplasm	−1.05	−1.06
C-X-C motif chemokine ligand 2	*CXCL2*	230101_at	Extracellular space	−5.50	2.98
Cytochrome P450 family 4 subfamily A member 11	*CYP4A11*	211231_x_at	Cytoplasm	−4.69	−6.86
Epidermal growth factor receptor pathway substrate 8	*EPS8*	202609_at	Plasma membrane	1.01	1.07
Forkhead box O3	*FOXO3*	231548_at	Nucleus	3.55	1.44
KH domain-containing, RNA-binding, signal transduction-associated 1	*KHDRBS1*	200040_at	Nucleus	1.07	1.20
Ladinin 1	*LAD1*	216641_s_at	Extracellular space	3.78	−2.69
Lysosomal-associated membrane protein 2	*LAMP2*	226671_at	Plasma membrane	−1.35	1.44
Lectin, galactoside-binding soluble 1	*LGALS1*	216500_at	Extracellular space	1.12	−1.40
LYN proto-oncogene, Src family tyrosine kinase	*LYN*	202625_at	Cytoplasm	−1.54	−2.42
Mitogen-activated protein kinase kinase kinase 2	*MAP3K2*	221695_s_at	Cytoplasm	−1.44	−1.80
Mitogen-activated protein kinase 7	*MAPK7*	35617_at	Cytoplasm	−1.21	1.11
Protein inhibitor of activated STAT 4	*PIAS4*	212879_x_at	Nucleus	−4.28	−4.52
Phosphoinositide-3-kinase regulatory subunit 1	*PIK3R1*	212239_at	Cytoplasm	−1.02	−1.24
Phosphoinositide-3-kinase regulatory subunit 5	*PIK3R5*	227553_at	Cytoplasm	−2.65	−8.48
Phosphatase and tensin homolog	*PTEN*	242622_x_at	Cytoplasm	3.91	49.96
Ras-related GTP binding B	*RRAGB*	205540_s_at	Cytoplasm	1.16	1.33
Succinate dehydrogenase complex iron sulfur subunit B	*SDHB*	214166_at	Cytoplasm	2.47	1.35
SH2 domain containing 2A	*SH2D2A*	207351_s_at	Cytoplasm	−2.67	1.40

[a]Normalized ratio of fold change of signal at 6 and 12 h of storage to corresponding signal of control group.

Supplementary Table 2. Amino acid composition of hBM-MSCs stored in PBS

Amino acid	Control (n = 3)	PBS-incubated group (n = 3) Signal[b]			Normalized value[a]	
		6 h	12 h	*p* value	6 h	12 h
Alanine	3.3	4.7	3.9	0.1259	1.4	1.2
Glycine	3.2	5.4	4.7	0.0026	1.7	1.5
Valine	3.0	3.2	2.8	0.0165	1.1	1.0
Leucine	4.9	5.4	4.9	0.0752	1.1	1.0
Isoleucine	5.2	5.4	4.7	0.0767	1.0	0.9
Threonine	6.7	3.0	3.7	0.0857	0.4	0.6
Serine	11.2	11.7	14.5	0.0410	1.1	1.3
Proline	3.4	4.4	3.6	0.0450	1.3	1.1
γ-Aminobutyric acid	0.0	0.1	0.1	0.0019	2.4	3.8
Pyroglutamic acid	36.8	30.4	14.4	0.0014	0.8	0.4
Phenylalanine	2.0	2.4	2.3	0.2681	1.2	1.1
Aspartic acid	1.2	0.9	0.8	0.1338	0.8	0.7
Glutamic acid	3.6	3.1	4.9	0.0023	0.9	1.4
Asparagine	0.5	0.6	0.7	0.0648	1.1	1.3
Glutamine	1.8	1.1	1.5	0.0196	0.6	0.8
Lysine	0.5	0.8	2.1	0.0007	1.7	4.6
Tyrosine	12.8	17.4	30.3	0.0002	1.4	2.4

[a]Compared to control after 6 or 12 h of storage.

[b]Amino acid signal intensity.

Supplementary Table 3. Ingenuity Pathway Analysis-based profiles of lipid peroxidation-related genes in hBM-MSCs cells overtime

Entrez Gene Name	Symbol	Affymetrix ID	Location	Signal (fold change)[a]	
				6 h	12 h
acetylcholinesterase (Yt blood group)	*ACHE*	210332_at	Plasma Membrane	-5.09	-8.53
alpha-1-microglobulin/bikunin precursor	*AMBP*	214425_at	Extracellular Space	-4.55	-10.51
apolipoprotein B	*APOB*	223579_s_at	Extracellular Space	1.25	-5.34
apolipoprotein E	*APOE*	203382_s_at	Extracellular Space	-5.12	-7.97
complement component 3a receptor 1	*C3AR1*	209906_at	Plasma Membrane	2.31	4.04
cytochrome P450, family 2, subfamily E, polypeptide 1	*CYP2E1*	209976_s_at	Cytoplasm	5.85	-7.23
cytochrome P450, family 4, subfamily A, polypeptide 11	*CYP4A11*	211231_x_at	Cytoplasm	-4.69	-6.86
fibroblast growth factor receptor 2	*FGFR2*	211400_at	Plasma Membrane	4.66	-15.37
hemopexin	*HPX*	39763_at	Extracellular Space	-4.06	4.08
huntingtin	*HTT*	202390_s_at	Cytoplasm	-1.25	7.11
insulin receptor	*INSR*	226450_at	Plasma Membrane	-3.58	3.41
low density lipoprotein receptor	*LDLR*	217103_at	Plasma Membrane	-21.51	-11.90
microtubule-associated protein tau	*MAPT*	203930_s_at	Plasma Membrane	-8.08	-3.53
NADPH oxidase 4	*NOX4*	236843_at	Cytoplasm	-7.65	-6.11
paraoxonase 1	*PON1*	206344_at	Extracellular Space	-2.27	3.33
peroxiredoxin 3	*PRDX3*	209766_at	Cytoplasm	1.85	-9.27
protein kinase, AMP-activated, alpha 2 catalytic subunit	*PRKAA2*	238441_at	Cytoplasm	11.52	22.26
presenilin 1	*PSEN1*	1559206_at	Plasma Membrane	-2.64	9.57
prostaglandin E receptor 3 (subtype EP3)	*PTGER3*	210375_at	Plasma Membrane	-17.23	27.73
prostaglandin E receptor 4 (subtype EP4)	*PTGER4*	204896_s_at	Plasma Membrane	1.21	8.43
superoxide dismutase 2, mitochondrial	*SOD2*	215078_at	Cytoplasm	-2.25	-7.44
tocopherol (alpha) transfer protein	*TTPA*	210614_at	transporter	2.02	3.71

[a]Normalized ratio of fold change of signal at 6 and 12 h of storage to corresponding signal of control group.

Supplementary Table 4. RT-PCR primer sequences for genes encoding ROS related genes

Gene Name	Symbol	NCBI Ref. seq	Direction	Primer sequence (5'-3')
v-akt murine thymoma viral oncogene homolog 2	*AKT2*	AA769075	Forward	GGT ACT TCC TGC TGA AGA GC
			Reverse	AAC GGG TGC CTG GTG TTC TG
mitogen-activated protein kinase kinase kinase 2	*MAP3K2*	AF239798.1	Forward	CCT TCA GTT CCC CAG ACC AG
			Reverse	ATT CCG GGC AAC CTG GTG
phosphoinositide-3-kinase, regulatory subunit 1 (alpha)	*PIK3R1*	NM_001242466.1	Forward	CGA CAG ATG GAC AGT GTG AC
			Reverse	CCA AGA GCT ACT AAG GAC CC
forkhead box O3	*FOXO3*	NM_201559.2	Forward	CAT GCG GGT CCA GAA TGA GG
			Reverse	CTA GAG CTC CGC TGC ATG AG
KH domain containing, RNA binding, signal transduction associated 1	*KHDRBS1*	NM_006559.1	Forward	CAG CCT CGG TCA AGA TGG AG
			Reverse	CAC GTC CAC GAG AGG GTT CA
Homo sapiens glyceraldehyde 3-phosphate dehydrogenase	*GAPDH*	NM_002046	Forward	CAT GAC CAC AGT CCA TGCCAT CAC T
			Reverse	TGA GGT CCA CCA CCC TGT TGC TGT A

Ref. seq.: Reference sequence

Supplementary Table 5. qPCR primer sequences for genes encoding ROS related genes

Gene Name	Symbol	NCBI Ref. seq	Direction	Primer sequence (5’-3’)
v-akt murine thymoma viral oncogene homolog 2	*AKT2*	AA769075	Forward	CTG AAT GAG AGG GAG TGG TTC
			Reverse	TGG GTC TGT ACT GGA ATT TGG
mitogen-activated protein kinase kinase kinase 2	*MAP3K2*	AF239798.1	Forward	CAG TGG AGA AGG CTA TGG AAG
			Reverse	GGC TGA GTG GCG ATT TTA AAG
phosphoinositide-3-kinase, regulatory subunit 1 (alpha)	*PIK3R1*	NM_001242466.1	Forward	GTC ATA CTG TCA CTG CTC TGG
			Reverse	TGA ATG AGA AGG ACT GCC AC
forkhead box O3	*FOXO3*	NM_201559.2	Forward	ATA GGC AAA AGG AGT GGA GC
			Reverse	CAT TAG CTG AGG ACA CTG ACA G
KH domain containing, RNA binding, signal transduction associated 1	*KHDRBS1*	NM_006559.1	Forward	CGA GTG CTG ATA CCT GTC AAG
			Reverse	GAG CCC TTT CCC AAT ACA GAG
Homo sapiens glyceraldehyde 3-phosphate dehydrogenase	*GAPDH*	NM_002046	Forward	GAA GAC TGT GGA TGG CCC
			Reverse	CCA TGC CAG TGA GCT TCC

Ref. seq.: Reference sequence

Supplementary Table 6. qPCR primer sequences for genes encoding lipid peroxidation related genes

Gene Name	Symbol	NCBI Ref. seq	Direction	Primer sequence (5'-3')
Homo sapiens superoxide dismutase 2	*SOD2*	BC016934.1	Forward	TGA GCC ACA TTC CGT TAC AC
			Reverse	GAC CAA ACA TTT CCC CAA AGC
Homo sapiens cytochrome P450, family 4, subfamily A, polypeptide 11	*CYP4A11*	BC041158.1	Forward	TGT TTG ACC CTT TCC GTT TTG
			Reverse	GAC TTC CCT CAT TCC TCT ATT CG
Homo sapiens tocopherol (alpha) transfer protein	*TTPA*	BC058000.1	Forward	AGG TAG AAA CTC AGC GGA ATG
			Reverse	GGC TAC GGA TGG AGT GAT TTG
Homo sapiens prostaglandin E receptor 4 (subtype EP4)	*PTGER4*	BC101534.1	Forward	ATC TTA CTC ATT GCC ACC TCC
			Reverse	TGA CTT CTC GCT CCA AAC TTG
Homo sapiens protein kinase, AMP-activated, alpha 2 catalytic subunit	*PRKAA2*	BC069823.1	Forward	TGT CTT CAG TTT CAC CTC GC
			Reverse	AGA CAG ATC AAC GGG CTA AAG
Homo sapiens glyceraldehyde 3-phosphate dehydrogenase	*GAPDH*	NM_002046	Forward	GAA GAC TGT GGA TGG CCC
			Reverse	CCA TGC CAG TGA GCT TCC

Ref. seq.: Reference sequence

Supplementary Table 7. RT-PCR primer sequences for genes encoding adipocyte specific marker genes

Gene Name	Symbol	NCBI Ref. seq	Direction	Primer sequence (5’-3’)
Homo sapiens peroxisome proliferator activated receptor gamma	*PPARγ*	NM_015869.4	Forward	TGT TAT GGG TGA AAC TCT GGG
			Reverse	GAT ATC AAA GGA GTG GGA GTG G
Homo sapiens complement factor D	*Adipsin*	NM_001317335.1	Forward	GGT CAC CCA AGC AAC AAA GT
			Reverse	CTC CTG CGT TCA AGT CAT C
Homo sapiens glyceraldehyde 3-phosphate dehydrogenase	*GAPDH*	NM_002046	Forward	GAA GAC TGT GGA TGG CCC
			Reverse	CCA TGC CAG TGA GCT TCC

Ref. seq.: Reference sequence

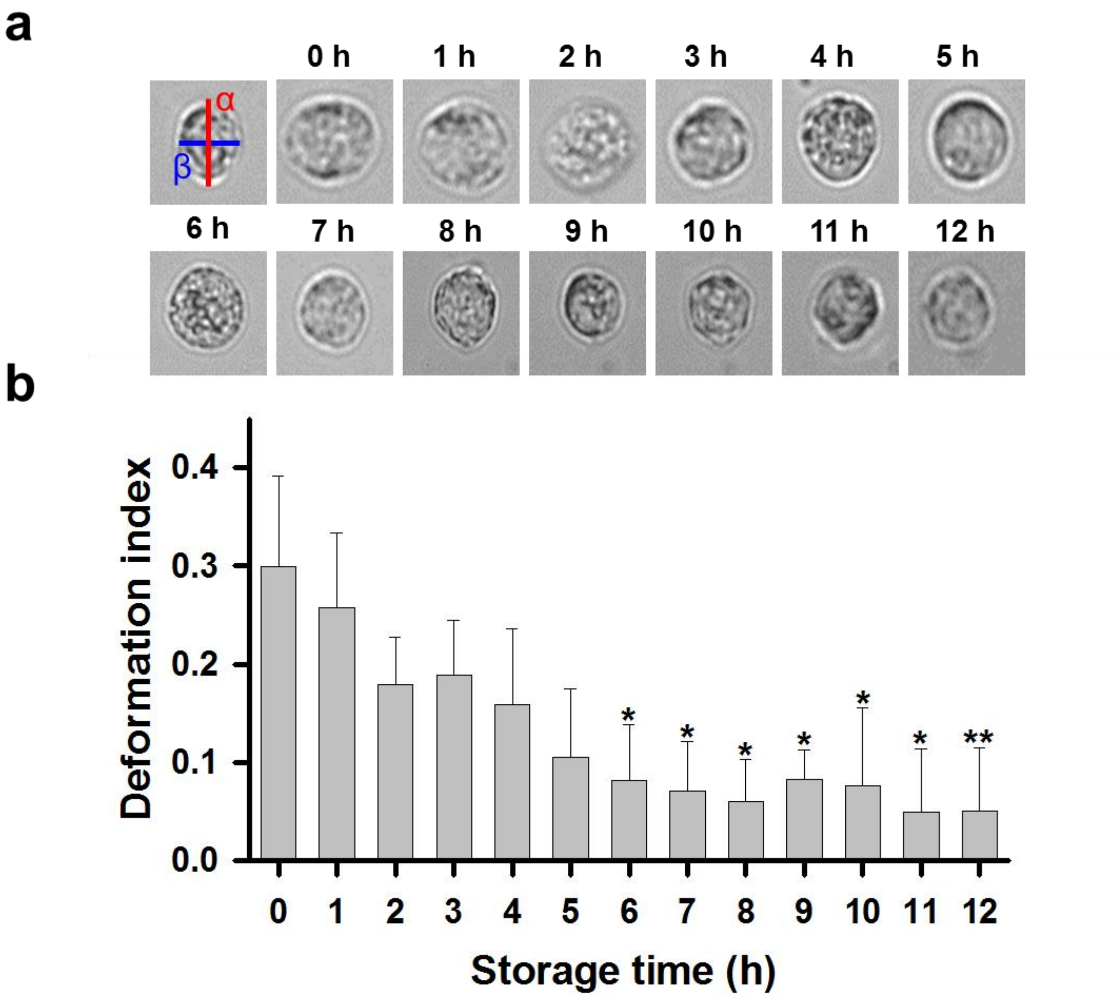

Supplementary Figure 1. Changes in deformability of hBM-MSCs stored in PBS. (a) Representative images for measuring deformation index of cells. Alpha; longest length of cell, beta; shortest length of cell. (b) Hourly changes in deformability in stored hBM-MSCs. Data are represented as the mean ± SD. *P < 0.05 *vs*. 0 h (control), **P < 0.01 *vs*. 0 h (control).

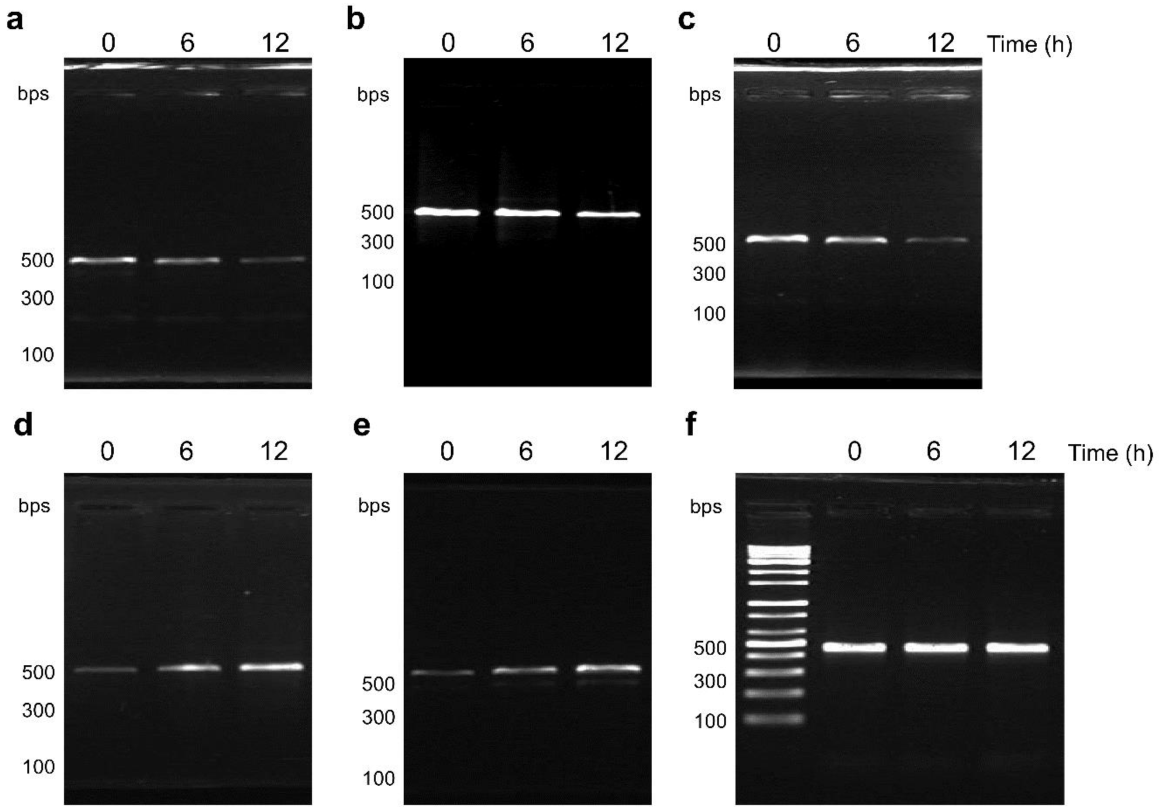

Supplementary Figure 2. Original gel images of RT-PCR for the ROS-related genes *AKT2* (a), *MAP3K2* (b), *PIK3R1* (c), *FOXO3* (d), *KHDRBS1* (e), and *GAPDH* (f) for Figure 2**b**. hBM-MSCs were stored in PBS for 6 and 12 h, and total RNA was reverse transcribed into cDNA. RT-PCR was performed using gene-specific primers.

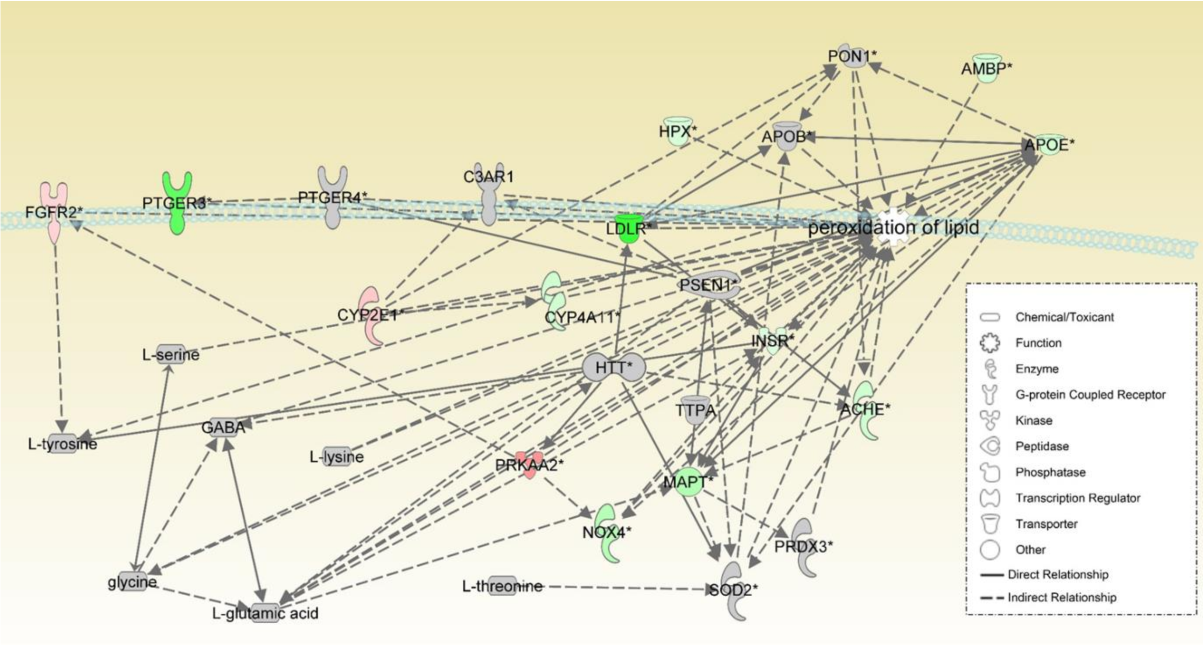

Supplementary Figure 3. Bioinformatics analysis of microarray and amino acid profiles in hBM-MSCs, stored in PBS for 6 h. Lipid peroxidation related gene and amino acid network were constructed algorithmically by IPA. Red and green areas indicate up and downregulated genes, respectively. Differentially expressed genes obtained from microarray data (genes with > 3-fold change) are shown.

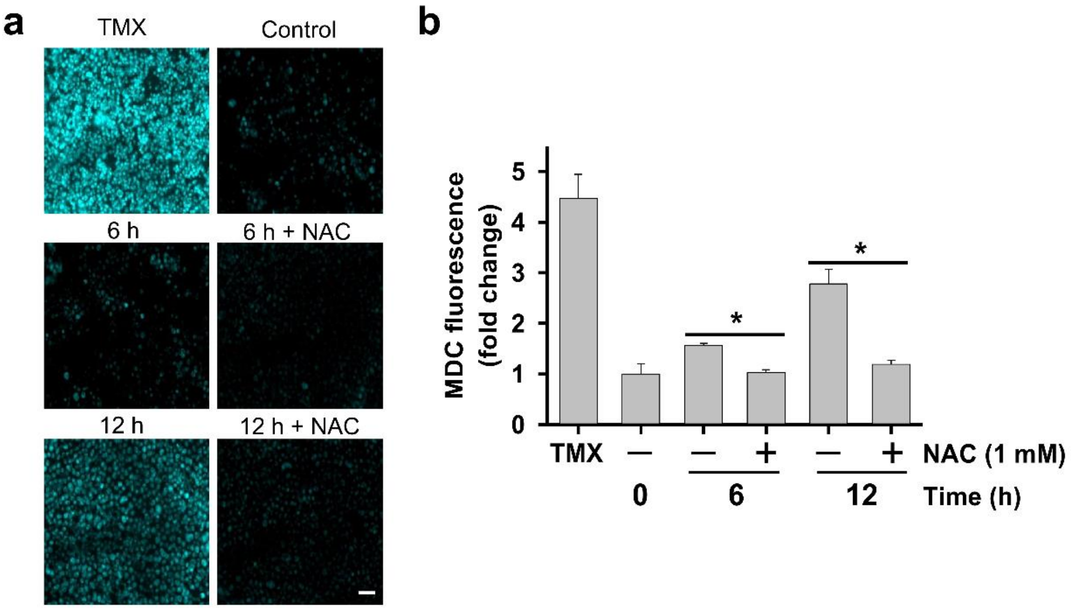

Supplementary Figure 4. Analysis of autophagy induction in stored hBM-MSCs. (a) Representative images for MDC staining of incubated hBM-MSCs. Tamoxifen (TMX) was used as positive control for autophagy. Scale bar = 100 μm. (b) Evaluation of autophagy induction in 6 h or 12 h-incubation in hBM-MSCs using MDC and fluorescence microplate reader at 335/512 nm (excitation/emission). The intensities are normalized with 0 h control. Data are represented as the mean ± SD of three independent experiments. *P < 0.05 *vs*. 0 h (control).

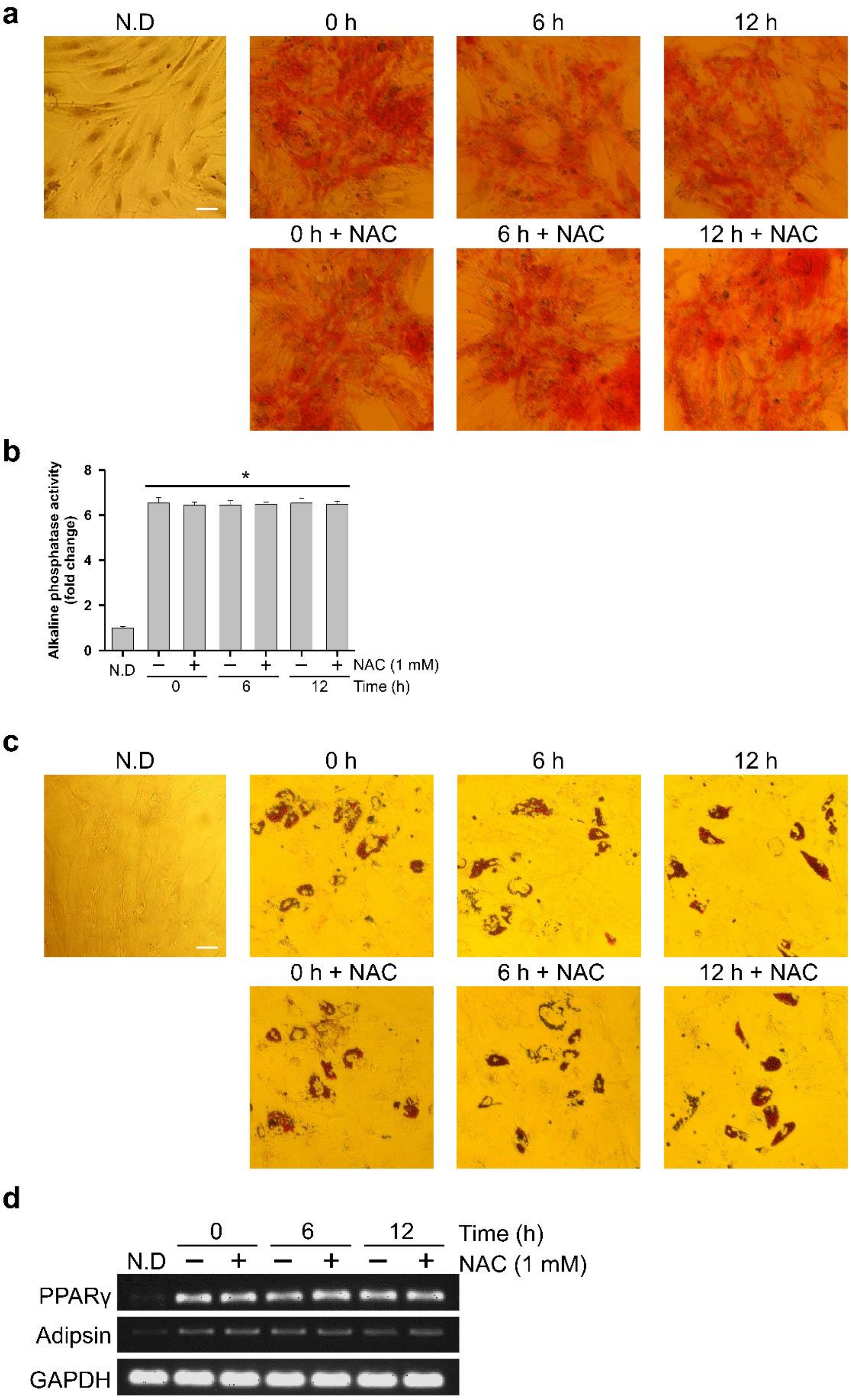

Supplementary Figure 5. Evaluation of osteogenic and adipogenic potentials in stored hBM-MSCs. (a) Images of mineral deposits in the osteogenic differentiated hBM-MSCs after storage stained with Alizarin Red S. The mineralization is stained as red. (b) The activity of

alkaline phosphatase was estimated after differentiation. (c) Images of lipid deposits in the adipogenic differentiated hBM-MSCs after storage stained with Oil Red O. Lipid droplets of adipocyte were stained as red. (d) Semi-quantitative RT-PCR detection of adipocyte specific marker genes (*PPARγ* and *Adipsin*) in hBM-MSCs after differentiation. Glyceraldehyde 3-phosphate dehydrogenase (*GAPDH*) served as an internal control. Bands were cropped from Supplementary Fig. 6. Scale bar = 20 µm. N.D, nondifferentiated control. Data represent mean ± SD of three independent experiments. *$P < 0.05$

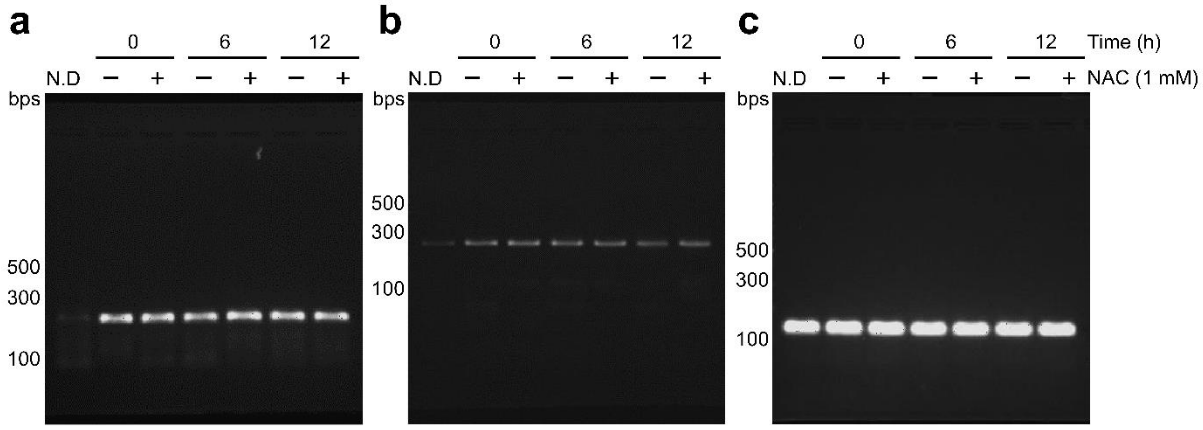

Supplementary Figure 6. Original gel images of RT-PCR for the adipocyte specific marker genes *PPARγ* (a), *Adipsin* (b), and *GAPDH* (c) for Supplementary Figure 5**d**. hBM-MSCs were stored in PBS for 6 and 12 h, and then were adipogenic differentiated for 2 weeks. Total RNA was reverse transcribed into cDNA. RT-PCR was performed using gene-specific primers. N.D, nondifferentiated control.

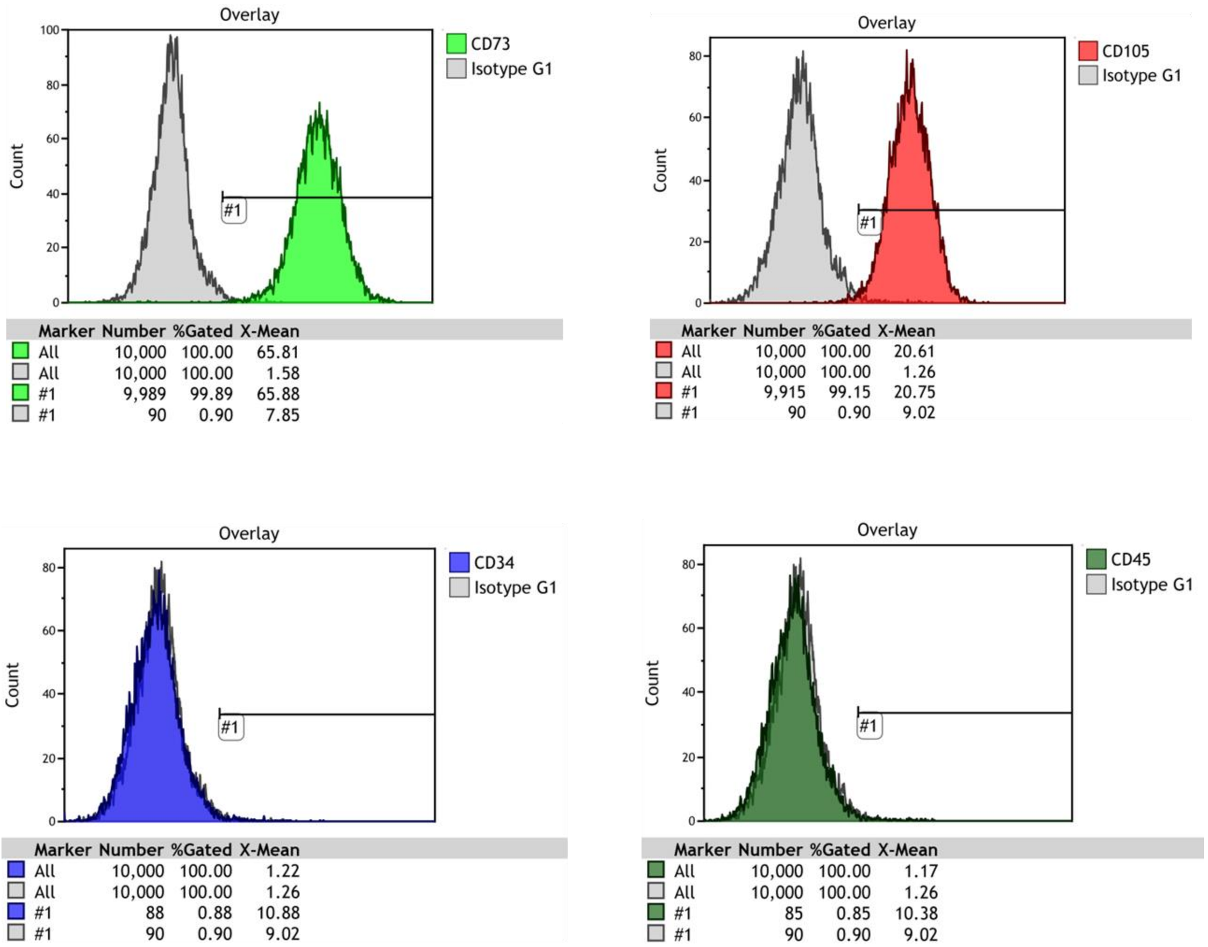

Supplementary Figure 7. Characteristics of hBM-MSCs. Flow cytometric analysis of hBM-MSCs surface markers, CD73 and CD105 (upper panel). Negative markers of hBM-MSCs, CD34 and CD45 (lower panel).
